# Monitoring and Predicting Treatment Response of Extraocular Muscles in Grave's Orbitopathy by ^99m^Tc-DTPA SPECT/CT

**DOI:** 10.3389/fmed.2021.791131

**Published:** 2021-12-16

**Authors:** Chengzhi Jiang, Zilong Deng, Jin Huang, Haoyu Deng, Jia Tan, Xinhui Li, Min Zhao

**Affiliations:** ^1^Department of Nuclear Medicine, Xiangya Hospital, Central South University, Changsha, China; ^2^Department of PET-CT Center, Hunan Cancer Hospital/The Affiliated Cancer Hospital of Xiangya School of Medicine, Central South University, Changsha, China; ^3^Department of Ophthalmology, Xiangya Hospital, Central South University, Changsha, China; ^4^National Clinical Research Center of Geriatric Disorders, Xiangya Hospital, Central South University, Changsha, China

**Keywords:** Grave's ophthalmopathy, extraocular muscles, single-photon emission computed tomography/computed tomography, glucocorticoid, treatment response

## Abstract

**Objective:** To investigate single-photon emission computed tomography/computed tomography (SPECT/CT) for assessing inflammation in the extraocular muscles (EOMs) and predicting the therapeutic efficacy of periocular glucocorticoid therapy (PGT) for Grave's ophthalmopathy (GO).

**Materials and Methods:** A total of 412 eyes from 206 patients with GO referred for ^99m^Tc-DTPA orbital SPECT/CT were enrolled. Fourteen age- and gender-matched healthy controls (28 eyes) were included. The thickness and uptake ratio (UR) of four EOMs were derived from SPECT/CT. Eighty-six eyes from patients with GO patients received PGT. Changes in SPECT/CT parameters were evaluated between the pre- and post-treatment.

**Results:** 195 eyes and 217 eyes were classified as active and inactive stages by clinical activity score (CAS). Values of the thickness and UR of each EOM, T_max_, and U_max_ were all significantly higher in the active GO than in the inactive GO and controls (*p* < 0.01). Among the 86 eyes (48 GO patients) included in the efficacy analysis, 56 eyes and 30 eyes were classified as responders and non-responders. Values of thicknesses and UR of each EOM, the maximum thickness (T_max_), and the maximum UR (U_max_) all dropped following PGT in the responders (*p* < 0.01). Logistic regression analysis identified the U_max_ as an independent predictor for the responders (*p* < 0.01). Moreover, the U_max_ demonstrated incremental predictive value over clinical characters and CAS, as evidenced by the improved area under the curve (0.85 vs. 0.78) and global chi-square (34.12 vs. 18.1).

**Conclusion:** ^99m^Tc-DTPA SPECT/CT has the potential to assess inflammatory activity by detecting the involvement of EOMs in GO. Pre-treatment UR provides independent and incremental values for the prediction of PGT treatment response.

## Introduction

Grave's orbitopathy (GO) is the most common extrathyroidal manifestation of Grave's disease and one of the most prevalent orbital disorders in adults (1–3). Signs and symptoms include eyelid retraction, proptosis, motility restriction, exposure keratopathy, and even vision loss, associated with a significant decrease in the quality of life of patients. As an autoimmune disease, GO follows a two-stage process, with an active inflammatory stage followed by an inactive fibrotic stage (2, 3). Anti-inflammatory treatment is usually considered effective during the active stage, but it has little value during the inactive stage (1, 4). Therefore, an accurate and objective assessment of inflammatory activity is essential to determine the appropriate treatment for GO.

The clinical activity score (CAS), based on inflammatory signs and symptoms, has been widely used for GO evaluation and as a criterion and guideline for therapeutic management (1). However, the acute inflammatory involvement of EOM or orbital fat may fail to be adequately assessed, especially when diplopia or motility impairment is not present (5–8). Moreover, diplopia and strabismus can be induced by either inflammation in the active stage or fatty degeneration and fibrosis in the inactive stage. MRI is another useful modality for GO imaging by the nature of its superior soft tissue contrast and no ionizing radiation. Especially, T2-weighted images can assist in staging and deciding treatment. However, the overall accuracy is still limited (9). Thus, a more precise assessment of EOM inflammatory activity is needed.

Currently, ^99m^technetium (^99m^Tc)-labeled diethylene triamine pentaacetic acid (DTPA) orbital single-photon emission computed tomography/computed tomography (SPECT/CT) or SPECT has proven to be a valuable method for the detection of inflammation in GO (5, 10). Theoretically, DTPA is uniformly distributed throughout the extracellular space, binds to polypeptides in the extracellular fluid, and does not cross the blood-tissue barrier. The amount of ^99m^Tc-DTPA accumulation in the soft tissue of orbital cavity (mainly in the EOMs) is directly proportionate to the activity of the inflammation with associated hyperpermeability and breakdown of blood-tissue barrier (5, 11). This may explain the high uptake of ^99m^Tc-DTPA in GO due to inflammation. Moreover, it provides not only visual but also semi-quantitative information about the activity of the disease. Although DTPA SPECT/CT imaging in patients with GO has been validated in some cases (10, 11), a systematic evaluation of the DTPA uptake of EOMs has not been validated. The aim of our study was to examine the SPECT/CT parameters of EOMs for assessing the inflammatory activity of GO, and to evaluate the role in predicting the efficacy of treatment.

## Materials and Methods

### Study Population and Clinical Assessment

This study was approved by the Ethics Committee of Xiangya Hospital (No. 202101021). All the patients provided written informed consent for the imaging procedures as well as for participation in anonymized analyses. Data from 302 patients with GO who underwent orbital ^99m^Tc-DTPA SPECT/CT were retrospectively collected in a single center from November 2016 to May 2017. All the patients with GO were diagnosed based on Bartley's criteria (1). Ninety-six patients with orbital tumors, other orbital inflammatory lesions, sinusitis, and history of systemic GC or radiation therapy were excluded. Finally, the remaining 206 patients with GO (412 eyes) were enrolled in this study.

All the patients underwent a full ophthalmological examination. The inflammatory activity of the eyes was assessed using the seven-point modified formulation of the CAS (1). Fourteen healthy subjects (28 eyes) without ophthalmological disorders and systemic immune diseases served as the control group.

Among the patients with GO, we selected those who received periocular corticosteroid therapy (PGT) for efficacy analysis. Inclusion criteria included CAS ≥ 2 and elevated uptake of EOMs by SPECT/CT. Patients with corticosteroid contraindication and pregnant or lactating women were excluded in this study. A total of 86 eyes received a periocular injection of 20 mg triamcinolone acetonide (40 mg/ml) (Jida Corporation, Kunming, China) weekly for 7 consecutive weeks. The selection of injection was based mainly on the CAS score or lesion of high uptake on SPECT/CT images. If SPECT/CT had high DTPA uptake only in the superior rectus, the injection was applied at the superomedial quadrants; if high uptake was present only in the inferior rectus, the injection was applied at the inferolateral quadrants. When SPECT/CT uptake was high in several recti, the injection was applied alternately at the inferolateral and superomedial quadrants. All the patients were evaluated by the same experienced ophthalmologist and had a final examination 3 to 6 months after the treatment. Response was assessed and defined by at least one of the following criteria: (1) the CAS dropped by at least 2 points and CAS <3; (2) no residual uptake of EOMs was observed on the follow-up SPECT/CT images. Patients who did not fulfill the above criteria were classified as “non-responders.”

### Orbital ^99m^Tc-DTPA SPECT/CT Acquisition

All the subjects were scanned with a hybriddouble head SPECT/CT scanner (Precedence 16, SPECT/CT; Philips, Netherlands) using a low-energy and high-resolution collimator. After 20 min of intravenous administration of 555 MBq ^99m^Tc-DTPA (Chinese Atomic Energy Institute, Beijing, China), an orbital CT scan (140 kV, 100 mA, 1 slice thickness) for attenuation correction was obtained with the patient's head positioned parallel to the Frankfurt plane. Then, SPECT images were acquired with 64 projections in step-and-shoot mode over 360 degrees (5.6 degrees per step), and matrix size was 64 ×64. The energy window was open by ±10% centered at 141 keV. Subsequently, the CT and SPECT images were loaded into an EBW workstation (Philips, Netherlands) for further analysis.

### SPECT/CT Imaging Measurement

**Figure 1:**
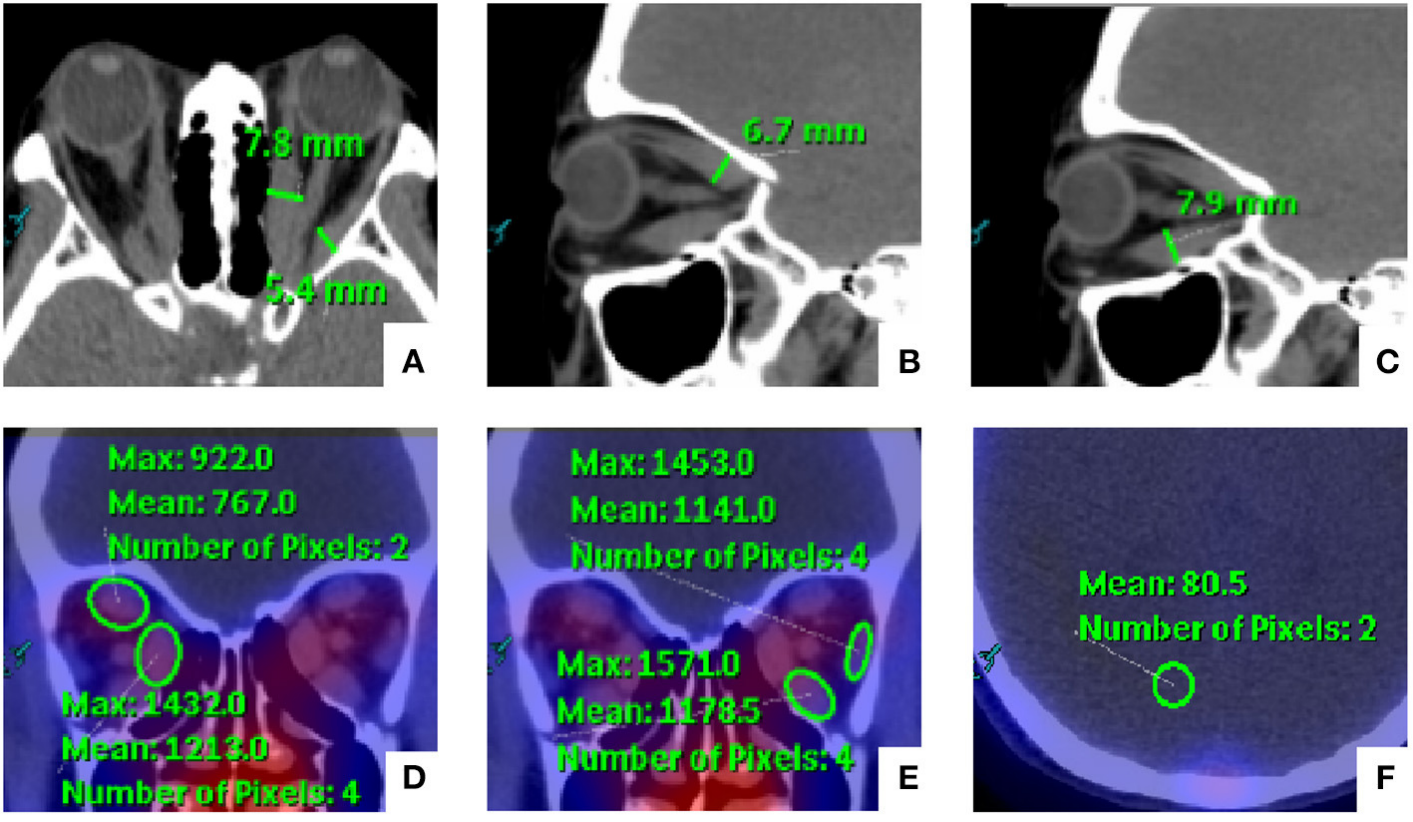
Schematic diagram for the measurement of orbital single-photon emission computed tomography/computed tomography (SPECT/CT) semi-quantitative parameters of extraocular muscles (EOMs). **(A–C)** The medial rectus (MR) and lateral rectus (LR) were measured on the axial images, and the vertical diameters of the superior rectus (SR) and inferior rectus (IR) were measured on the sagittal images. In addition, **(D,E)** the EOM uptake values were determined by manually placing a round region of interest (ROI) within the consensus highest uptake portion of each EOM on the CT attenuation-corrected SPECT images. **(F)** The background uptake value was similarly determined through the analogous placement of an ROI on the occipital lobe.

Manual rigid registration of SPECT and CT images was carried out on the EBW workstation. Two experienced nuclear medicine specialists (MZ and CZJ) who were blinded to the SPECT/CT results evaluated the orbital SPECT/CT images together. The thickness and DTPA uptake of EOMs were evaluated by the two readers. Horizontal diameters of the medial rectus (MR) and lateral rectus (LR), and the vertical diameters of the superior rectus (SR) and inferior rectus (IR) were measured on the series of images. In addition, the DTPA uptake was determined by manually placing a round region of interest (ROI) with the consensus highest uptake of each EOM on the CT attenuation-corrected SPECT images. The background uptake value was similarly determined through analogous placement of an ROI on the occipital lobe based on our previous studies (12). The methods for SPECT/CT parameter measurement are illustrated in Figure 1. For the same patient, the value of uptake ratio (UR) was calculated as the ratio of the maximum EOM uptake value to the maximum background uptake value. Furthermore, we chose the highest value of thickness and UR among the four EOMs as the maximum thickness (T_max_) and the maximum UR (U_max_).

### Statistical Analysis

Continuous values were expressed as mean ± SD. The comparison among groups of continuous variables was performed by Student's *t*-test, Mann–Whitney U test, and one-way ANOVA depending on the nature of data. Categorical variables were presented as numbers and percentages and analyzed by Fisher's exact test. The univariate binary logistic regression analysis was applied to estimate potential predictors for the response of PGT. The multivariable binary logistic regression was performed to analyze the independent predictors, and variables with *p* < 0.05 in the univariate analysis were included. Moreover, the efficacy of treatment was evaluated by comparing the receiver operator characteristic (ROC) curve of binary logistic regression from different models. χ^2^ Statistic by a likelihood ratio test was performed to calculate the incremental value of UR. Intra- and inter-observer agreements of parameters were assessed by the respective intraclass correlation coefficients (ICCs) from 20 randomly selected sets of images. Statistical analysis was performed using the IBM SPSS 25.0 software (IBM Corp., Armonk, NY, United States). *P* < 0.05 was considered statistically significant.

## Results

### Comparison of EOM Parameters

According to the CAS, all the eyes were classified into the active GO (CAS ≥ 3/7, 104 patients, *n* = 195) and inactive GO (CAS <3/7, 115 patients, *n* = 217) groups. The average age of active GO was 46 ± 12 years, which was higher than that of the inactive GO and control groups (*p* < 0.01 and *p* < 0.05 respectively). There was a significant difference in gender and smoking history between active GO and inactive GO (*p* < 0.01 and *p* < 0.05 respectively). Active GO patients showed significant increased thicknesses and UR of each EOM and U_max_ than those in both the inactive GO patients and the controls (all *p* < 0.01). The thickness values for the MR, SR, IR, T_max_, UR of each EOM, and U_max_ in inactive GO were higher than those in the control group (*p* < 0.05). No significant difference in thickness value for the LR was found between the inactive and control subjects. The clinical characteristic and SPECT/CT parameters are summarized in Table 1.

**Table 1:** Extraocular muscles (EOM) measurements among the three groups.

**Characteristics**	**Active group**	**Inactive group**	**Control**	***p*-value**
	**(195 eyes)**	**(217 eyes)**	**(28 eyes)**	
Female, *n* (%)	95 (48.7)^##^	153 (70.5)^*^	16 (57.1)	0.000
Age (years)	46 ± 12^*^^##^	39 ± 12	41 ± 12	0.112
Smokers, *n* (%)	68 (34.9)^#^	56 (25.8)	12 (42.9)	0.171
CAS, points	3.6 ± 0.9^##^	1.4 ± 0.7	/	0.000
**Thickness, mm**				
LR	4.2 ± 1.2^**^^##^	3.5 ± 1.0	2.8 ± 0.6	0.000
MR	5.6 ± 2.0^**^^##^	4.3 ± 1.5^*^	3.2 ± 0.5	0.000
SR	4.9 ± 2.0^**^^##^	3.7 ± 1.5^*^	2.7 ± 0.7	0.000
IR	6.1 ± 2.0^**^^##^	4.6 ± 1.7^*^	3.3 ± 0.6	0.000
T_max_	6.9 ± 2.0^**^^##^	5.2 ± 1.7^**^	3.6 ± 0.5	0.000
**UR, no unit**				
LR	8.56 ± 1.99^**^^##^	7.32 ± 1.85^*^	6.82 ± 1.19	0.108
MR	11.63 ± 2.38^**^^##^	9.16 ± 1.96^*^	8.15 ± 1.34	0.001
SR	9.22 ± 3.18^**^^##^	7.29 ± 2.18^*^	6.08 ± 1.34	0.000
IR	11.06 ± 2.49^**^^##^	8.91 ± 2.05^**^	7.73 ± 1.24	0.001
U_max_	12.31 ± 2.27^**^^##^	9.76 ± 2.03^**^	8.39 ± 1.20	0.002

*CAS, clinical activity score; LR, lateral rectus; MR, medial rectus; SR, super rectus; IR, inferior rectus; T_max_, maximum thickness among the four EOMs; U_max_, maximum uptake ratio among the four EOMs*.

*Compare with the control group*,

* *P < 0.05*,

** *P < 0.01;*

*compareith the inactive group*,

# *P < 0.05*,

## *P < 0.01*.

### Outcomes and Effectiveness of PGT

Of the 86 eyes treated with PGT, 56 eyes (65.1%) exhibited response. At baseline, the responders had greater thickness of the LR and SR and higher UR of the LR, MR, IR and U_max_ than the non-responders (*p* < 0.05); whereas, sex, smoking status, serum TRAb, CAS, CAS staging, thickness of the MR and IR, T_max_, and UR of SR were similar between the two groups (all *p* > 0.05) (Table 2).

**Table 2:** Baseline EOM measurements.

**Characteristics**	**Responders**	**Non-responders**	***p*-value**
	**(56 eyes)**	**(30 eyes)**	
Female, *n* (%)	32 (57.1)	15 (50.0)	0.526
Age (years)	41 ± 12	47 ± 11	0.026
Smokers, *n* (%)	17 (30.4)	8 (26.7)	0.719
TRAb (IU/L)	14.51 ± 13.63	15.02 ± 11.24	0.871
CAS, points	3.1 ± 0.9	2.8± 1.0	0.240
CAS≥3, *n* (%)	40 (71.4%)	15 (50.0%)	0.061
**Thickness, mm**			
LR	3.8 ± 1.0	4.3 ± 1.0	0.027
MR	5.6 ± 2.3	5.1 ± 1.8	0.306
SR	4.3 ± 1.5	5.2 ± 1.8	0.026
IR	6.1 ± 1.8	6.3 ± 2.8	0.742
T_max_	6.7 ± 2.0	7.0 ± 2.5	0.539
**Uptake ratio, no unit**			
LR	8.31 ± 1.53	7.58 ± 1.42	0.035
MR	11.34 ± 2.08	9.65 ± 2.19	0.001
SR	9.02 ± 2.34	8.63 ± 2.30	0.465
IR	10.82 ± 1.88	8.92 ± 2.37	0.000
U_max_	11.93 ± 1.74	10.46 ± 2.07	0.001

*TRAb, thyroid-stimulating hormone (TSH) receptor antibodies; CAS, clinical activity score; LR, lateral rectus; MR, medial rectus; SR, super rectus; IR, inferior rectus; T_max_, maximum thickness among the four EOMs; U_max_, maximum uptake ratio among the four EOMs*.

**Figure 2:**
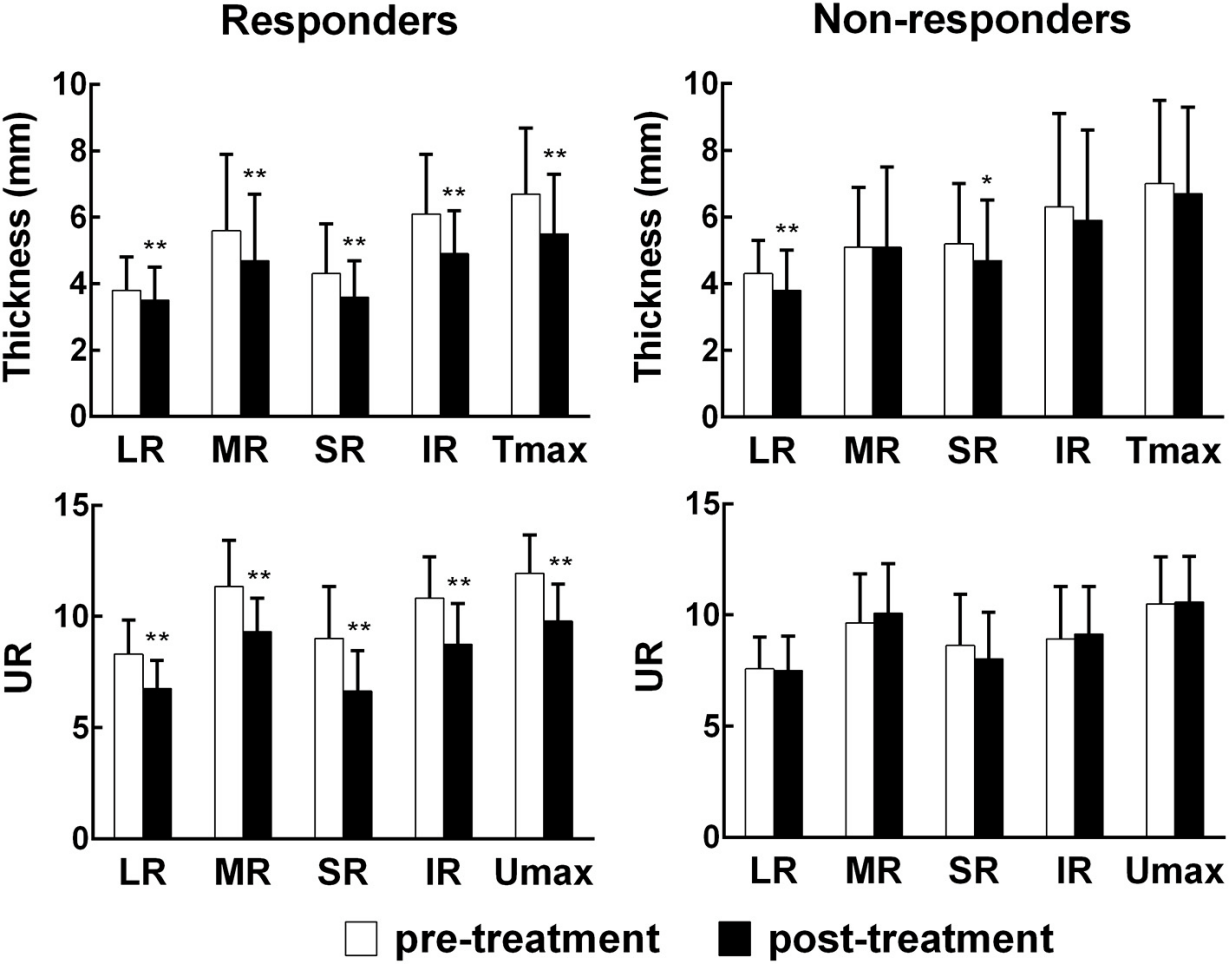
Comparisons of orbital SPECT/CT parameters before and after treatment. EOM, extraocular muscle; LR, lateral rectus; MR, medial rectus; SR, super rectus; IR, inferior rectus; T_max_, maximum thickness among the four EOMs; U_max_, maximum uptake ratio among the four EOMs.*p< *0.0*5, **p< *0.0*1, compared with the pre-treatment group.

Subgroup analyses were performed between pre-treatment and post-treatment (Figure 2). The thickness and UR of each EOM, T_max_, and U_max_ were reduced accordingly in the responders (all *p* < 0.01). In the non-responders, the thickness of the LR and SR decreased after PGT (*p* < 0.01, *p* < 0.05), but the UR of each EOM and U_max_ remained unchanged following PGT (all *p* > 0.05).

### Variables for Predicting the Response to PGT

Univariate and multivariate logistic regression analyses were further performed to identify the predictive factors of response following PGT, such as age, gender, smoking habit, TRAb, CAS, T_max_, and U_max_. In the univariate analysis, age (OR 0.96, 95% CI 0.92–1.00, *p* = 0.03) and U_max_ (OR 1.57, 95% CI 1.71–2.11, *p* = 0.002) were significantly associated with the responders. In the multivariate analysis, age (OR 0.91; 95% CI 0.86–0.96; *p* = 0.001) and U_max_ (OR 2.08, 95% CI 1.41–3.06, *p* < 0.001) remained the independent predictors of the responders. The results are shown in Table 3.

**Table 3:** Univariate and multivariate logistic regression analyses in predicting response to therapy.

**Variables**	**Univariate analysis**			**Multivariate analysis**		
	**OR**	**95%CI**	***P*-valve**	**OR**	**95%CI**	***P*-valve**
Age (years)	0.96	0.92–1.00	0.030	0.91	0.86–0.96	0.001
Female (%)	1.33	0.55–3.25	0.526			
Smoking (%)	1.20	0.45–3.23	0.720			
TRAb (IU/L)	0.99	0.95–1.02	0.439			
CAS ≥ 3(%)	0.40	0.16–1.01	0.051			
T_max_, mm	0.94	0.77–1.15	0.535			
U_max_, no unit	1.57	1.17–2.11	0.002	2.08	1.41–3.06	<0.001

*TRAb, thyroid-stimulating hormone (TSH) receptor antibodies; CAS, clinical activity score; T_max_, maximum thickness among the four EOMs; U_max_, maximum uptake ratio among the four EOMs*.

**Figure 3:**
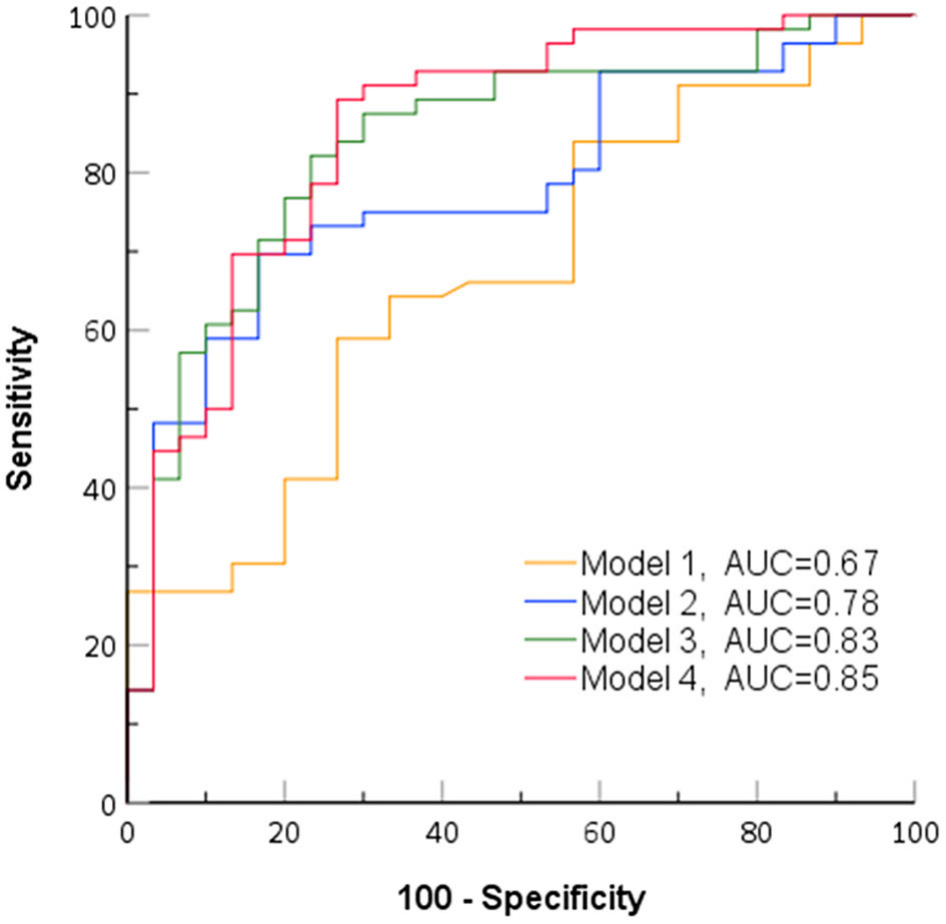
Receiver operating characteristic curves of identified models for predicting response to periocular glucocorticoid therapy (PGT). AUC, area under the curve; model 1, clinical characteristics; model 2, clinical characteristics + clinical activity score (CAS) ≥ 3; model 3, clinical characteristics + U_max_; model 4, clinical characteristics + CAS ≥ 3+ U_max_.

**Figure 4:**
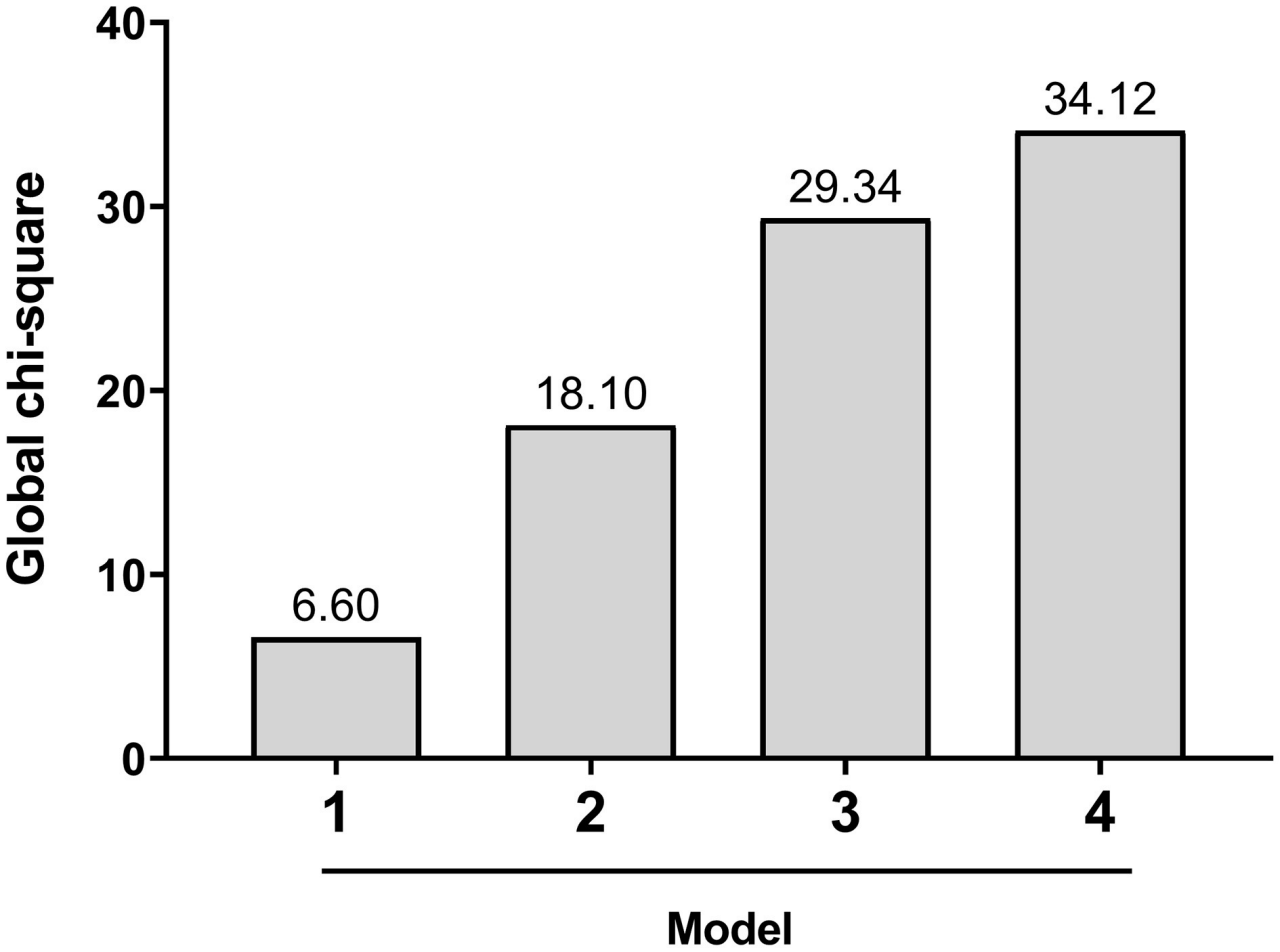
Incremental value of U_max_ for the prediction of responder to PGT. Model 1, clinical characteristic; model 2, clinical characteristics + CAS ≥ 3; model 3, clinical characteristics + U_max_; model 4, characteristics + CAS ≥ 3 + U_max_.

In the ROC analysis of predictive models (Figure 3), model 1, incorporating clinical characters, alone showed the lowest area under the curve (AUC) (sensitivity 0.64, specificity 0.66, AUC 0.67). The AUC of model 2 combining clinical characters and CAS ≥ 3 (sensitivity 0.75, specificity 0.70, AUC 0.78), model 3 combining clinical characters and U_max_ (sensitivity 0.82, specificity 0.73, AUC 0.82), and model 4 combining clinical characters, CAS ≥ 3, and U_max_ (sensitivity 0.89, specificity 0.73, AUC 0.85) increased sequentially. Furthermore, likelihood ratio tests indicated that both CAS ≥ 3 and U_max_ provided a significant incremental predictive value for PGT response (Figure 4). The addition of CAS ≥ 3 and U_max_ increased the global chi-square as compared to the clinical characters (6.60 vs. 18.1, 6.60 vs. 29.34, *p* < 0.001, respectively). The model 4 further yield the greater global chi-square when compared to the model 1, 2, and 3 (*p* < 0.001, *p* < 0.001, and *p* < 0.05, respectively).

### Reproducibility

The intra-observer reproducibility of measuring thickness and UR of EOMs were excellent, as reflected by high ICCs (0.985, 95% CI 0.981–0.989, *p* < 0.001;0.981, 95% CI 0.977–0.985, *p* < 0.001, respectively). Furthermore, the reproducibility of inter-observer was 0.971 (95% CI 0.965–0.981, *p* < 0.001) for thickness and 0.968 (95% CI 0.958–0.973, *p* < 0.001) for UR.

## Discussion

The notable results of this study were as follows: (1)we found significantly higher uptake and thickening of EOM evaluated by orbital SPECT/CT in patients with active GO than in patients with inactive GO. These parameters appeared useful for the differentiation disease activity between inactive and active GO; (2) the UR of EOMs as determined from SPECT/CT at baseline provided the independent and incremental values for the prediction of response following PGT.

To date, the CAS is the most commonly used clinical scale to determine the indication and duration of anti-inflammatory treatment. However, it presents some limitations that reflect ocular surface inflammation and has certain subjectivity. Orbital CT can provide information on exophthalmos and fat and muscle enlargement, which can be useful for diagnosis. The current study showed that the thickness of four EOMs in the active GO group were greater than those in the inactive GO group and control group, consistent with previous studies (13, 14). Additionally, we found that abdominal enlargement of the EOMs, especially the inferior and medial rectus muscles, was most common in GO. Although the thickness of the EOMs could reflect the stage of GO, the involvement of each EOM can occur in different phases, and it may be difficult to identify which EOM is in the inflammatory phase by clinical assessment alone. Therefore, it is challenging to evaluate the inflammatory activity from CT measurement.

Orbital ^99m^Tc-DTPA SPECT has been used to evaluate autoimmune inflammation of the retro-bulbar area in patients with GO for many years (5, 11). However, only the retro-orbital area of a SPECT image could be analyzed for the assessment of inflammation, without allowing for a more precise indication of which EOM is involved. In addition, physiological uptake in adjacent nasal sinuses can sometimes lead to falsely positive accumulation in the retro-orbital cavity. Thus, our study aimed to evaluate the EOMs of GO using a hybrid SPECT/CT method. SPECT/CT depiction of EOM inflammation may help clinicians to accurately evaluate the inflammatory staging of GO (10, 13). Our findings showed that the UR of EOM, especially the medial and inferior rectus muscles, in the active GO group was higher than that in the inactive GO group and the control group, suggesting that the UR has a potential for the evaluation of inflammatory infiltration within EOMs.

The European Group of Grave's Orbitopathy (EUGOGO) recommended systemic GC treatment for patients with moderate-to-severe GO as the first-line therapy (1, 15–18). However, medications can cause many side effects. Previous studies have found that periocular injections of GC were safe and effective in mild-to-moderate GO without causing serious complications (10). In clinical practice, the classification of clinical outcomes following GC therapy in GO has not been consistent: some patients with mild GO had significant improvements with local steroid therapy (5, 10), but a portion of patients with moderate-to-severe GO did not respond to systemic steroids (9, 19). This heterogeneity could be attributed to the presence of fibrosis and residual inflammation not detectable by current imaging modalities. Thus, it is essential to accurately evaluate the degree of inflammation and select the appropriate time for treatment.

In this study, we evaluated the predictive value of DTPA SPECT/CT for the efficacy of PGT in GO. Our results showed that the UR and thickness of EOMs all significantly dropped after treatment in the responders. However, in the non-responders, the UR of EOMs remained unchanged after treatment, even if the thickness of the superior and lateral rectus muscles decreased after the treatment. Additionally, it appeared that the UR of EOMs provided independent and incremental information for the prediction of response following PGT. These findings suggested that GO was likely to improve significantly with PGT in patients with high inflammatory burden at baseline.

What is also interesting is that this study suggests that initial CAS may not predict improvement following PGT. This can be explained by the fact that only patients with mild GO and elevated DTPA uptake, in spite of having low CAS (2–3 points), were included for analysis. This was because based on our previous study (10), patients with GO and CAS below 3 points might also benefit from PGT as long as the SPECT/CT showed DTPA activity in the eyes. Moreover, another reason is that CAS is judged on the basis of eye signs and symptoms in anterior segment, while SPECT/CT reflects activity in the posterior orbital segment, the major site of inflammation deposit. Thus, we speculated that patients with high uptake ratio of EOMs would, thus, be recommended to receive PGT, which would be superior to the conventional CAS score methods.

This study has several limitations. First, no uniform consensus regarding the definition of treatment response exists, and various factors (such as the CAS) complicate the objective evaluation of inflammatory activity. Second, partial volume effect remains one of the major degrading factors that hamper quantitative accuracy in SPECT imaging, particularly for small structures. Further studies using PET/CT will be needed to validate this finding, as PET/CT has a higher spatial resolution than SPECT/CT. Third, our study concerned only the predictability of DTPA SPECT/CT for the efficacy of periocular steroid treatment in patients with GO, and evaluation of the efficacy of systemic steroid treatment in patients with GO, thus, needs to be further investigated.

## Conclusion

Orbital ^99m^Tc-DTPA SPECT/CT provided a reliable and feasible technique for assessing the inflammatory activity of EOMs in patients with GO. The UR of EOMs could be used as an objective index for evaluating the therapeutic efficacy in patients with GO.

## Data Availability Statement

The original contributions presented in the study are included in the article/supplementary material, further inquiries can be directed to the corresponding authors.

## Ethics Statement

The studies involving human participants were reviewed and approved by Xiangya Hospital (No. 202101021). The patients/participants provided their written informed consent to participate in this study.

## Author Contributions

CZJ participated in study design and data analysis and interpretation, performed the statistical analysis, and drafted the manuscript. ZLD and JH collected the imaging data. HYD and JT contributed to editing and review of the manuscript. MZ and XHL contributed to study design and editing and review of the manuscript. All the authors read and approved the submitted version.

## Funding

This study was supported by the National Natural Science Foundation of China (No. 81901784 to MZ) and Hunan Provincial Clinical Technology Innovation Project (No. 2020SK53705 to MZ).

## Conflict of Interest

The authors declare that the research was conducted in the absence of any commercial or financial relationships that could be construed as a potential conflict of interest.

## Publisher's Note

All claims expressed in this article are solely those of the authors and do not necessarily represent those of their affiliated organizations, or those of the publisher, the editors and the reviewers. Any product that may be evaluated in this article, or claim that may be made by its manufacturer, is not guaranteed or endorsed by the publisher.
